# Dural Venous Sinus Thrombosis: A Case Report

**DOI:** 10.31729/jnma.7170

**Published:** 2021-12-31

**Authors:** Dhiraj Chaurasia, Bikash Yadav, Krishna Dhungana

**Affiliations:** 1 Kathmandu Medical College and Teaching Hospital, Sinamangal, Kathmandu, Nepal; 2 Department of Neuromedicine, Kathmandu Medical College and Teaching Hospital, Sinamangal, Kathmandu, Nepal

**Keywords:** *anticoagulants*, *neuroimaging*, *seizures*, *thrombosis*

## Abstract

Dural Venous Sinus Thrombosis is the formation of blood clot within the cerebral sinus. It is very rare case with varying clinical presentation. It has non-specific signs and symptoms ranging from headache, papilledema, seizures, focal neurological deficits and mental state changes which is caused by genetic and acquired prothrombotic states, infections, inflammatory disease and trauma. Magnetic Resonance Imaging with Magnetic Resonance Venography is the specific imaging technique for the diagnosis. We have described a case of a patient who presented with headache over the temporal and occipital region and was disoriented. The Computed Tomography, Magnetic Resonance Imaging, Magnetic Resonance Venography report revealed presence of thrombus in the transverse and sigmoid sinus with hemorrhagic infarcts. He was then treated with anticoagulants Low Molecular Weight Heparin which was further substituted by Warfarin.

## INTRODUCTION

Dural Venous Sinus Thrombosis (DVST) refers to the formation of blood clot in the cerebral sinus within the venous lumen that affects all the age groups.^1^ It is rare with an estimated incidence of about 3 to 4 per million cases in adults and about 7 cases per million among neonates and children.^2,3^ The major pathogenesis behind development of DVST is increased intracranial pressure due to impaired venous drainage and focal neurodeficit from venous infarcts or haemorrhage.^4^ We report a case of 29 years old male who presented with severe headache and diagnosis of transverse and sigmoid sinus thrombosis.

## CASE REPORT

A 29-years-old male, presented in Emergency department of Kathmandu Medical Hospital and Teaching Hospital with complaints of headache for 2 days and responding with inappropriate answers whenever questioned. The headache was sudden in onset in left temporal and occipital region, throbbing type, gradually progressive since hours. The patient had experienced jerk in Zipline in an amusement park in Nepalgunj. He, then went to a tertiary hospital with complaints of severe headache on left side, memory loss and blurred vision, with loss of 72 hours of knowledge. Disorientation was present and reaction time was decreased, gives irrelevant answers and could not identify his own family members. The thought stream was continuous. On examination, general condition was fair. There was no signs of pallor, icterus, lymphadenopathy, clubbing, edema or dehydration. The body temperature was 97°F, pulse 74 beats per minute, respiratory rate 20 times per minute, blood pressure 130/80 mmHg, oxygen saturation of 98% measured with pulse oximeter. His systemic examination was normal. Glassgow Comma Scale had score of E4, V5, M6. High mental function shows negative meningeal sign/neck rigidity. Kernig sign was negative. On cranial nerve examination, the pupil showed bilateral 3mm reactive and cerebral sign were normal. The motor examination and reflex response were normal. The sensory response was intact.

The investigations were done on intervals (Table 1).

**Table 1:** Investigation reports of the patient.

Parameters	2021		
	03/16	03/20	03/25
Hb (g/dl)	13.0	13.2	
TC (/mm3)	6900	8100	
DC (/mm3)	N60L32	N65L25	
Platelets (/mm3)	138000	135000	
PT/INR	9/1.0	10/1.1	15/1.66
Fasting Blood Sugar (mg/dl)	111	100	
Urea/Creatinine (mg/dl)	29/1.0	23/1.0	
Na/K (mmol/l)	140/3.8	138/4.0	

**Figure 1:**
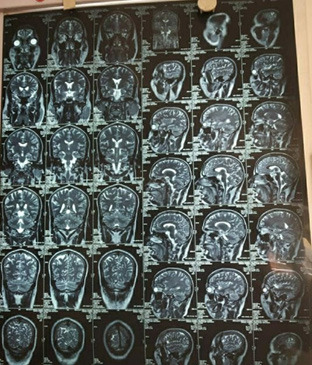
MRI of brain showing T2 and flair images showing left subacute thrombus noted in transverse sinus.

Thrombophilia Comprehensive profile as reported showed lupus like anticoagulant present. The Patient value/Control value was 49.4/37.2. The antithrombin activity was found to be 90%. Protein C value was 106. Protein S, Free antigen value was 123. The ultrasound abdomen and pelvis showed right nephrolithiasis and mild fatty liver changes. The Electroencephalogram was within normal limit and was slow for age. The Magnetic Resonance Imaging (MRI) of the brain showed left subacute thrombus noted in left transverse sinus extending up to internal jugular vein (Figure 1).

**Figure 2:**
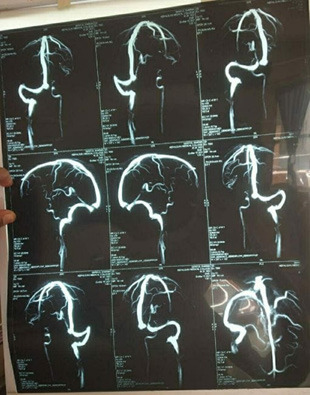
MRV of brain showing left transverse and sigmoid sinus thrombosis.

Subacute infarction of the left parietooccipital watershed area hemorrhage which transform likely to venous infarction. The Non-Contrast Computed Tomography (NCCT) of the brain showed left transverse and sigmoid sinus thrombosis (Figure 2) leading to venous infarction with hemorrhagic transformation in the left parietal region.

**Figure 3:**
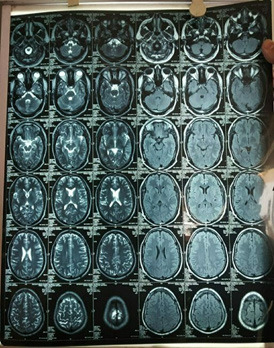
MRI of brain showing T2 sagittal and coronal images showing subacute infraction in parietoccipital region.

MRI of the brain shows the subacute infraction in parietoccipital region (Figure 3).

The patient was admitted in Intensive Care Unit (ICU) for 3 days and was latter shifted to the cabin for 7 days. Low molecular weight heparin was started and then anticoagulation was done with warfarin. Patient developed Pneumonia during the hospital stay which was managed with antibiotics (Clavum, Dalacin) and other supportive medications were done. As noted from investigations report the was recovering well and was found to be in normal state of health. So, discharge was planned. At the time of discharge, he was prescribed with medicine (Table 2).

**Table 2:** Medicines and its dosages prescribed during discharge.

Medication	Dose	Route of administration	Times	Duration
Dalacin	300mg	Per Oral	4 times a day	4 days
Clavum	625mg	Per Oral	Three times a day	4 days
Farin	6mg	Per Oral	Daily (6PM)	To continue
Levocet	5mg	Per Oral	At bedtime	5 days
Levera	500mg	Per Oral	Twice a day	To continue
B One	100mg	Per Oral	Three times a day	1 month
Pantocid	40mg	Per Oral	Daily	7 days
Codopar	1 Tab	Per Oral	If necessary	

The patient was then discharge with an advice for follow-up in Neuromedicine OPD after 7 days with PT/INR and Chest X-ray report.

## DISCUSSION

DVST is one of serious neurological condition which comprises (1-3%) of all strokes.^5^ The most common sinus affected in DVST are the superior sagittal sinus (70-80%) followed by transverse and sigmoid sinus (70%).^6^ Here, in our present case the patient develops thrombosis in the transverse and sigmoid sinus with presence of infarcts in the parietooccipital vein.

The major etiological risk factors for identified cases includes genetic and acquired prothrombotic states, pregnancy, puerperium, infections (sinusitis, mastoiditis, meningitis), oral contraceptives, trauma, dehydration, inflammatory diseases, etc.^1-3^ In young women, sinus thrombosis is found to occur more commonly during the puerperium than during pregnancy.^7^ The genetically induced prothrombotic states includes Antithrombin III, protein C and S deficiency, mutation in factor V, prothrombin (20210GA) and homocysteinemia. The acquired prothrombotic states causing DVST includes nephrotic syndrome, hyperhomocysteinemia, and antiphospholipid antibodies.^2,8^ Direct head injury to sinus or jugular vein are the mechanical causes of DVST.^1^ In our case, the causative factor for developing DVST is possibly head trauma which causes injury to dural sinus and extended up to jugular veins.

The most common presenting symptoms in cases of DVST includes headache (88.8%), seizures (39.3%), paresis (28.3%) and mental state changes (22%).^9^ Similar to this case, our patient presented with throbbing type, gradually progressive headache. Headache is recognized as most common symptoms which of sudden onset, develops over few hours or few days.^10^ Sometimes the headache of DVST can mislead mimicking migraine, subarachnoid hemorrhage with cluster like headache and even thunderclap headache.^11^ Headache are progressively increasing intensity. Seizures are more common in DVST than in arterial stroke cases. Focal neurological deficits consist paresis, dysarthria and aphasia which occurs due to venous infarction and damage in cerebral cortex. Papilledema can cause diplopia, orbital pain, blurring of vision and vision loss. Mental state changes such as amnesia, mutism, confusion occurs due thrombosis of deep system. In the neonates and children, the symptoms are almost similar to adults but neurological deficits and seizures are more common.^12^

Laboratory investigation and neuroimaging helps in reaching the diagnosis of DVST and further treatment. Lab investigation includes blood and serum profile, complete thrombophilia checkup and D-Dimer value.^1,2^ Neuroimaging techniques includes Computed Tomography (CT)/Computed Tomography Venography (CTV), Magnetic Resonance Imaging (MRI)/Magnetic Resonance Venography (MRV), Cerebral Angiography.^1^ CT shows triangle sign, cord sign or empty delta sign which shows brain edema and hemorrhagic infarcts.8 MRI along with MRV is the definitive imaging technique for DVST. In our case, MRI, MRV and NCCT were done. The results of the imaging were analyzed to come to a diagnosis. MRI along with MRV shows thrombus, absence in reduction of flow which helps in differentiating if it is hypoplastic sinus, partial sinus occlusion ,thrombosis of cortical veins or filling defects.^12^

The treatment option for DVST mainly includes general supportive measures, anticoagulation and endovascular thrombolysis.^3^ Anticoagulation administration is the prerequisite in the management of DVST. It can be of two types: i) aPPT; adjusted subcutaneous heparin and ii) body weight adjusted subcutaneous Low Molecular Weight Heparin(LMWH). These helps in arresting thrombotic process. Oral anticoagulants such as warfarin is then substituted with aim to keep International Normalized Ratio (INR) between 2 to 3.^8^ Similarly, endovascular thrombolysis can be done with administration of thrombolytic enzymes such as Urokinase or streptokinase. The use of thrombolytic agents were reported from few case reports and non-randomized clinical trials.^12^ In our case, the anticoagulation was done with body weight adjusted Low Molecular Weight Heparin and then was switched to Warfarin. The INR value was progressively increasing towards normal and the patient was recovering well. There was complication of pneumonia which was managed with antibiotics in the hospital stay. The recovery rate of patient with DVST was found to be 80% with varying time period of several weeks or months to be in normal state.^10^ Seizures and new thrombotic events were reported to be most common complication during follow-up.^9^

Thus, our case emphasizes on thorough imaging investigation to diagnose a rare entity as dural venous sinus thrombosis.
